# Clinical and radiographic evaluation of fendermate and T-band matrix systems for restoration of class II cavities in primary molars: a randomized clinical trial

**DOI:** 10.1186/s12903-026-07895-6

**Published:** 2026-03-18

**Authors:** Noha El-Sayed Fathi Abdou, Hitham Refat Ramadan Said, Asmaa Ali Emam Abo-Elsoud, Yousra Samir Helmy

**Affiliations:** 1 Lecturer at Pediatric Dentistry Department, Preventive Dentistry and Dental Public Health Faculty of Dentistry, Aswan University, Aswan city, Egypt; 2 Lecturer of Oral Radiology at Oral Radiology Department, Faculty of Dentistry, Suez Canal University, Ismailia city, Egypt; 3 Department of Pediatric Dentistry, Preventive Dentistry and Dental Public Health, Faculty of Dentistry, Suez Canal University, Ismailia city, Egypt

**Keywords:** Class II restorations, FenderMate, T-band, Primary molars

## Abstract

**Background:**

Restoring posterior primary teeth may result in issues such as improper contact points and proximal overhangs. New matrix systems were implemented to restore proper tooth contours and contacts.

**Aim of the study:**

This study aimed to assess and compare the radiographic and clinical effectiveness, patient comfort, and operator satisfaction in Class II cavity restoration in primary molars utilizing the FenderMate matrix system versus the T-band.

**Methods:**

This randomized clinical trial involving (20 children, 40 molars), ranging between 5 and 9 years, with bilateral class II cavities in primary molars (*n* = 40), indicated for class II restoration. Primary molars were randomized into two groups based on the matrix system used; Group I underwent the FenderMate system, whereas Group II underwent the T-band system. We assessed the clinical and radiographical efficacy of restorations, patient comfort, operator ease and satisfaction by the end of the procedure. Data were collected, and subjected to stastical analysis utilizing the chi-square test, The Mann-Whitney U test and, the Fisher exact test with statistical significance determined at *p* < 0.05.

**Results:**

The Optimum proximal contact was significantly achieved in 80% of T-band cases versus 30% with FenderMate (*p* = 0.006). No substantial differences were detetected between groups regarding radiographic proximal overhangs (*p* = 0.26) and ease of application/removal during primary molars’ class II cavity restoration (*p* = 0.11 and *p* = 0.12, respectively). However, the FenderMate group experienced significant gingival trauma (*p *< 0.001) and restoration dislodgment (*p *< 0.001) but required significantly less time for application (*p* < 0.001). The T-band group exhibited significant patient comfort (*P* = 0.02, 0.0001), while FenderMate caused significant patient discomfort (*P *= 0.0002).

**Conclusion:**

T- band matrices yield better proximal contacts and patient confort, while FenderMate provides greater time efficiency. These findings might assist clinicians in matrix selection for pediatric Class II restorations.

**Trial registration:**

This current controlled trial was registered at clinicalTrials.gov (ID NCT06447324) on June 10, 2024.

## Introduction and background

Dental caries is a multifactorial, localized, preventable, and transmissible disease that arises from an interplay of host factors, microflora, and dietary influences on the tooth surface over time, leading to cavitation in dentin and enamel. The primary bacteria associated with its etiology are Streptococcus mutans, responsible for its onset, as well as Lactobacilli species, which contribute to its progression. [1, 2]

The objective of Pediatric dentistry is to preserve primary teeth until the eruption of permanent successors. Primary teeth play a vital role in maintaining arch length, facilitating effective chewing, speech, and aesthetics while also preventing the development of abnormal oral habits. [3] Pediatric dentistry seeks to maximize benefits while minimizing intervention, particularly in addressing the challenges associated with treating young children who frequently exhibit fear of dental procedures. This necessitates that the dentist carry out the treatment with efficiency and speed. Untreated dental caries may lead to pain, infections, and challenges in chewing and can disrupt a child’s growth and development. Therefore, restoring primary teeth is crucial for their preservation until natural exfoliation occurs. [4, 7]

Proximal carious lesion progression in primary molars is considerably quicker than in permanent teeth. Therefore, restoring the tooth to its optimal shape and function is essential following the loss of tooth structure. [8, 9] The restoration of ideal contact and contour minimizes food impaction, protects the interdental gingival papilla, preserves a healthy periodontium, and stabilizes the dental arch by maintaining the normal mesiodistal relationship between teeth. Improper proximal contact or contour is associated with rotated or malaligned teeth, lifting forces and displacement leading to food lodgment, which can cause halitosis, initiation of dental caries, and periodontal diseases [10, 11]. 

The prevalence of improper contact area construction in primary teeth is a notable concern in pediatric dentistry as it can influence the development of approximal caries and malocclusion. Studies have shown that variations in the contact areas between primary molars are significantly associated with the risk of approximal caries. One prospective cohort study of children observed that approximal caries developed in about 13% of contacts over three years, indicating that improper or overly tight contact areas can encourage plaque accumulation and decay in primary teeth [12]. In terms of malocclusion and improper occlusal relationships which may be related to contact construction issues, studies indicate a high prevalence of malocclusion in primary dentition, ranging from about 43% to over 66% in different populations [13].

Thus, a significant challenge that dental clinicians face in Class II cavity restoration is achieving physiologically and anatomically accurate proximal contours and contacts [14, 15], specially for primary teeth, which exhibit a distinct cervical constriction and possess flatter and broader contact areas compared to permanent teeth. Matrix system placement is more challenging for primary teeth due to the increased likelihood of slippage. Furthermore, children exhibit limited attention spans and demonstrate unpredictable behavior, which hinders their potential to remain seated for extended periods [16]. 

Matrix bands are essential for achieving this required natural proximal contact, as they serve as pseudo walls during proximal carious lesion restoration placement. [17, 18] A matrix is a precisely shaped piece of metal or other material utilized to shape and support the restoration during its placement and setting process [19]. 

Clinical dental matrix systems for deciduous teeth mainly consist of two types: circumferential matrices and sectional matrices. Circumferential matrices are pre-shaped, three-dimensional bands specifically designed for pediatric teeth. They are used in combination with anatomical wedges and separators to ensure tight proximal contacts. These matrices typically provide better patient comfort and improved visibility.While circumferential matrices are generally reportedly preferred by children for comfort during Class II restorations, they may produce less optimal contact points compared to sectional matrices [15].

Sectional matrices: are anatomically contoured bands that improve restoration retention and adaptation to tooth form. Pediatric versions have a smaller height (around 5.5 mm) tailored to primary teeth. Sectional matrices, such as the Composi-Tight 3D system, tend to create more ideal proximal contacts but may require longer placement time and cause more discomfort for pediatric patients compared to circumferential matrices. Another example is the myJunior sectional matrix system, specially developed for smaller primary and young permanent teeth, offering thin steel bands and child-friendly components to facilitate conservative and comfortable treatment [15].

A recent systematic review, in 2025 aimed to evaluate the effectiveness of circumferential matrix band (CMB) and sectional matrix band (SMB) systems in obtaining optimum proximal contact in class II composite restorations. This study found the SMB to be more effective than the CMB in achieving optimal proximal contact in class II posterior composite restorations. Sectional matrices with separation rings produced significantly tighter contacts. Although operator satisfaction was similar for both systems, the sectional matrix was deemed easier to use [20].

A recent study by Gigova et al. (2025) investigated how the matrix system and filling material affect the proximal wall shape and contact tightness in Class II restorations of primary molars. They found that the type of matrix system has a greater impact on the effectiveness of the proximal contour in primary tooth restorations than either the filling material or the filling technique. Additionally, using sectional matrix systems specifically designed for primary teeth (such as the MuJuniorKit) significantly enhances the proximal contacts of Class II composite restorations in primary molars. In contrast, circumferential matrices are more likely to cause edge formation than sectional matrices [21].

A novel matrix band system utilizing shape memory polymer (SMP) has recently been developed. This system features two reversible shapes: a digitized permanent shape that replicates the anatomical proximal contour, and a programmable temporary shape designed for clinical application and restoration. The permanent shape can be customized using software or retrieved from an imaging database compiled from a large general population. However, there is currently no solid scientific evidence supporting the effectiveness of this newly introduced technique [22].

Other common pediatric matrix band systems include the T-band, ProMatrix, and FenderMate. These have shown good clinical efficacy in restoring proximal contacts and contours in primary molars [18].

The T-band is the most widely utilized matrix system for class II restorations in pediatric dentistry [18]. Numerous matrix, wedge, and separation techniques have been developed and improved. The FenderMate is a pre-shaped, single-piece sectional matrix and wedge, thus eliminating the necessity for separation rings, tailored for primary dentition. It is designed to enable quick, safe, and reliable composite restorations. It ensures proximal restoration with a tight contact and a secure seal at the cervical margin. This matrix can be inserted either from the buccal or lingual side. It extends from the wedge’s base up to just above the occlusal surface. The wedge side that faces the adjacent tooth features an angled wing, which presses the matrix firmly against the tooth preparation during insertion, creating a tight cervical seal. To help shape the contact point, the matrix includes a pre-formed indentation that follows natural tooth contours. [18]

An in-vitro study, on typodont teeth, compared various matrix systems, including sectional systems like Palodent V3, which shares a similar design concept with FenderMate sectional pre-contoured matrices, consistently produced accurate proximal contours and contact anatomy for Class II restorations. They demonstrated superior adaptation in the middle and occlusal thirds of the restorations and were more effective at reducing gingival overhang compared to many circumferential matrices [23].

Currently, dentistry lacks an adequately manufactured matrix for direct restorations specially for primary teeth. Various matrix systems offer distinct advantages as asserted by their manufacturers; nevertheless, there is a lack of sufficient evidence to substantiate these claims. This study aimed to evaluate the FenderMate matrix systems regarding their effectiveness in establishing optimal contact points, as well as operator ease, satisfaction, and patient comfort for class II restorations in primary molars, in comparison to the T-band, which the most widely utilized matrix system in pediatric dentistry for class II restorations. The objectives included the clinical assessment of Proximal Contact Points (PCPs) using waxed dental floss and proximal contours, aided by digital bitewing radiographs. Operator ease and satisfaction were evaluated through a detailed questionnaire, while patient comfort was assessed using the Wong-Baker pain rating scale.

The null hypothesis : there would be no significant difference in the effects of the FenderMate matrix system compared to the T-band matrix regarding PCPs, proximal contours, operator ease and satisfaction, and patient comfort in the restoration of proximal Class II primary molars.

The alternative hypothesis : there would be significant difference in the effects of the FenderMate matrix system compared to the T-band matrix regarding PCPs, proximal contours, operator ease and satisfaction, and patient comfort in the restoration of proximal Class II primary molars.

## Materials and methods

**Fig. 1:**
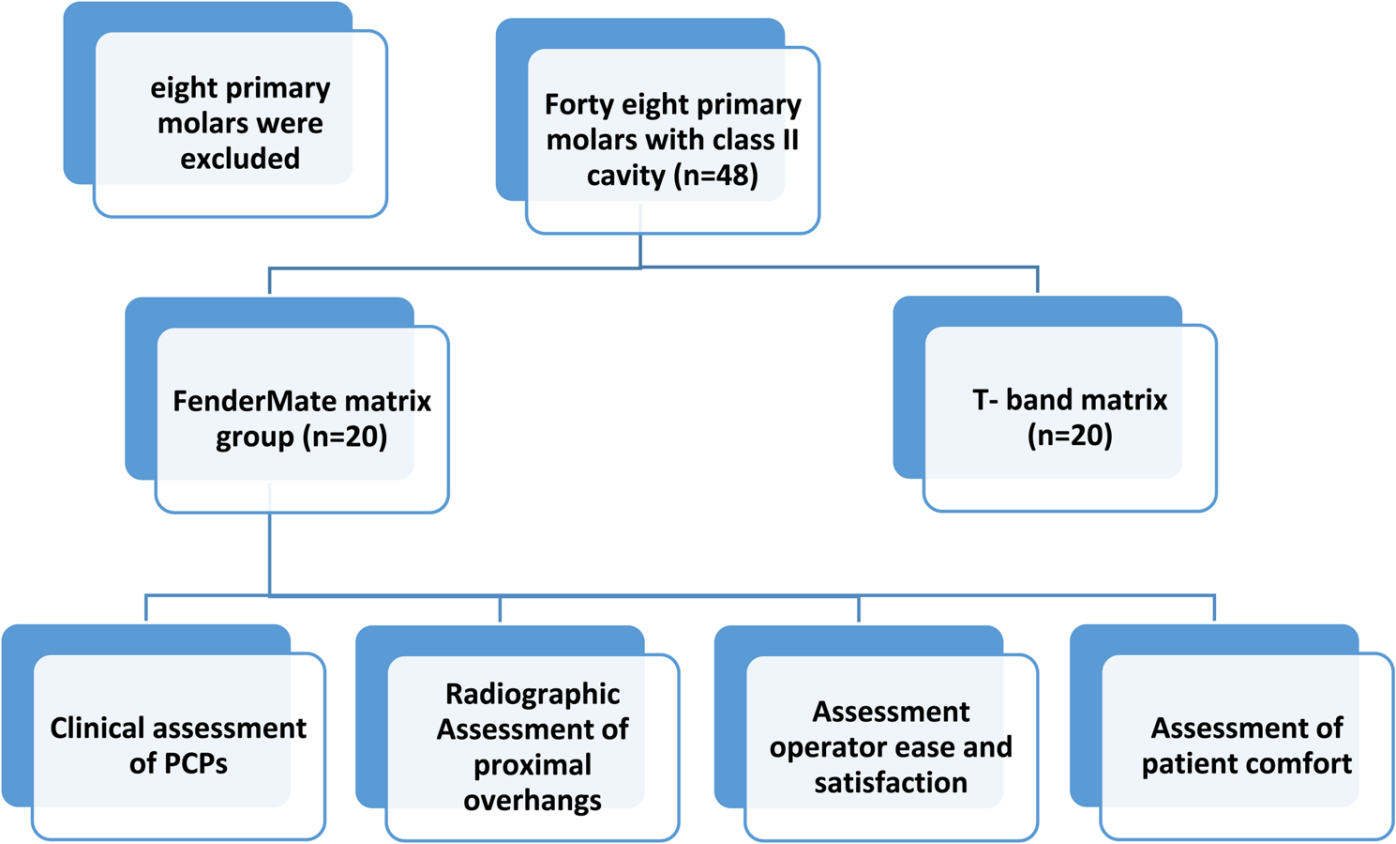
CONSORT flow diagram of the study

The current prospective, single blinded (by the examiner and observers), randomized, controlled clinical trial included 20 apparently healthy cooperative children (both sexes), aged 5–9 years, with 40 carious primary molars with bilateral Class II cavities. The children were selected from the Outpatient Clinic of the Pediatric Dentistry Department, Faculty of Dentistry Suez Canal University, following the agreement of the Faculty of Dentistry, Suez Canal University’s Research Ethics Committee (788/2024), and Registered on ClinicalTrials.gov under identifier number (NCT06447324). The trial was an interventional study utilizing a randomized parallel model assignment. The current study adheres to CONSORT guidelines. (Fig. 1) Since all the patients included in this study were under 16 years old, informed consent forms, regarding the nature of the treatment administered, following a comprehensive explanation of all procedures and potential complications, were signed by their parents or legal guardians.

Sample size calculation was performed using G*Power version 3.1.9.2, University Kiel, Germany. Copyright (c) 1992–2014 [24].The effect size W was 0.445 (large) according to the previous studies [18] based on digrees of freedom (d.f = 1) for chi square test (Goodness-of-fit tests: Contingency table) with alpha (α) level of 0.05 and Beta (β) level of 0.05, i.e., power = 80%; the estimated sample size (n) should be 40 samples and will be divided equally into 2 groups (20 samples/group).

Twenty children participating in this study underwent radiographic and clinical examinations utilizing digital bitewing radiographs to confirm that the bilateral Class II caries were confined to enamel and dentin, with adjacent teeth fully erupted. No history of mobility issues, pain, tenderness to percussion, or swelling was detected. Another crucial consideration was that the children must be cooperative and classified as “definitely positive” or “positive” based on Frankl’s behavior rating scale.16 Children with partially or ectopically erupted primary molars, children with special health care needs, and those uncomfortable with the rubber dam placement were excluded from the study [15].

### Randomization and grouping

Forty primary molars in twenty children were randomly allocated as follows:

One operator carried out all the procedures, with help from a clinical assistant who drew chits. The first chit determined whether the right or left side would be treated first, and the second chit decided which matrix system would be applied initially to prevent any bias.

- FenderMate matrix group (*n* = 20): Following the FenderMate Prime matrix (Dentistry Directa, USA) placement, primary molars were restored with composite restoration.
- Conventional T-band matrix group (*n* = 20): After placing the T-band matrix (Pulpdent, USA), primary molars were restored with a composite restoration.

### Clinical procedures

Following the verification of radiographic and clinical eligibility criteria, primary molars were isolated utilizing a rubber dam. The split dam technique was used when restoring multiple teeth in one quadrant. The technique involves creating a trough and stretching the rubber dam over several teeth in that quadrant. The rubber dam was secured at either end using clamps [25]. The preparation of disto-occlusal or mesio-occlusal cavities was confined to the extent of the caries and did not exceed the following dimensions: the proximal cavity measured 3 × 3.5 × 1.5 mm in the bucco-lingual, occluso-gingival, and mesio-distal directions, respectively. The occlusal cavity dimensions were 2.5 × 2 × 2.5 mm in the bucco-lingual, occluso-pulpal, and mesio-distal directions, respectively [26]. Any cases exceeding these dimensions were excluded.

The cavity preparation was done utilizing a single operator employing a high-speed diamond round bur (no. 01-Dentsply Maillefer-Tulsa-Oklahoma- USA) with water coolant. A new diamond round bur was used for each patient. Children experiencing pain throughout carious dentin excavation were administered local anesthesia. [15, 16]

**Fig. 2:**
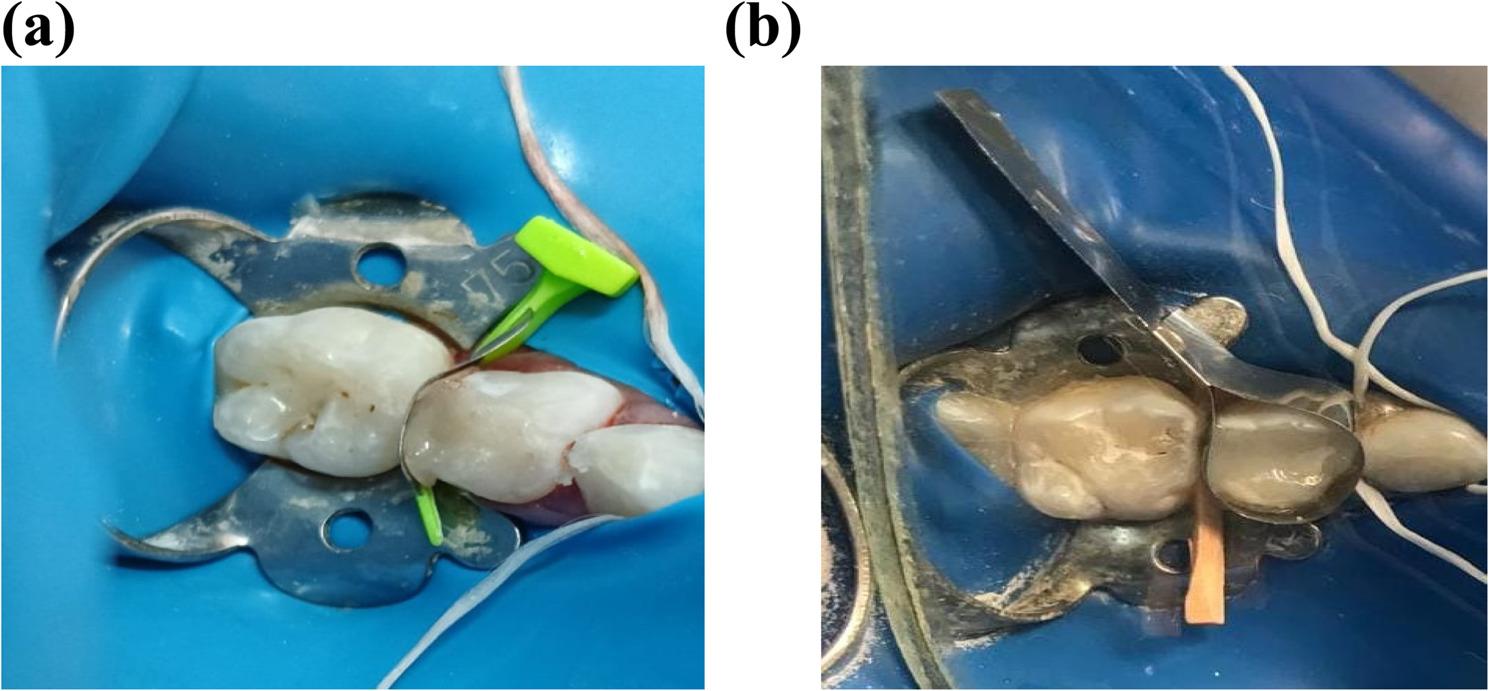
The matrix systems used: (**a**) FenderMate matrix system, (**b**) T-band matrix

In the FenderMate matrix group, the matrix was positioned lingually or buccally prior to placing the restoration. The matrix extends from the base of the wedge to a position just above the occlusal surface. The wedge’s side that faces the adjacent tooth features an angled wing. During insertion, the wing exerts pressure on the matrix, ensuring a secure seal at the cervical margin. The matrix features a pre-contoured indentation that replicates natural contours and is tailored to the tooth’s shape. In contrast, the conventional T-band matrix group utilized a matrix and wooden wedge inserted interproximal (Fig. 2).

Selective enamel etching was carried out using a 37% phosphoric acid gel (3 M™ Scotchbond™ Universal Etchant Etching Gel, 3 M ESPE, St Paul, USA) for 15 s, following the manufacturer’s guidelines. Any remaining acid was carefully rinsed off to ensure the etchant was fully removed. After thoroughly drying the tooth, the adhesive (3 M ESPE Scotchbond Single Bond Universal Adhesive, 3 M ESPE, St Paul, USA) was applied with a micro-brush and rubbed onto the surface for 20 s. It was then gently air-dried for about 5 s to evaporate the solvent, followed by light curing for 10 s as per the manufacturer’s instructions. The curing process was performed using an Elipar Deep Cure LED curing light (3 M ESPE, St Paul, USA) with an output irradiance of 1,470 mW/cm², appropriate for polymerizing light-curable dental materials activated by photo initiators within the 430–480 nm wavelength range. [26]

Composite resin restorations (Filtec bulk fill-3 M ESPE-USA) (Table 1) were applied using various matrix systems based on the group’s allocation. The restorations were light-cured once for 20 s from the occlusal side, matrix bands were initially removed, succeeded by the removal of wedges and the rubber dam. Restorations were finished occlussally using fine grit diamond burs (FG 257F023,FG166C010, Horico) and Sof-LexTM discs and the occlusion was checked with articulating paper (Bausch Articulating Paper [15, 16]. The proximal surfaces were not finished in order not to affect the contact tightness [27].

**Table 1:** Commercial names, type and composition of restorative materials used

Restorative material	Type	Composition	Shade	Manufacture
Filtek Bulk Fill Posterior Restorative	Full-body bulk-fill composite	Aromatic UDMA, UDMA, silica, DDDMA, silane treated ceramic, pentanedioic acid, 2,2-dimethyl-4-methylene-reaction products with glycidyl methacrylate, EDMAB, benzotriazol, titanium dioxide Filler loading: 76.5 wt%, 58.4 vol%	A1	3 M ESPE-USA

## Methods of evaluation

### PCP clinical assessment 

An examiner blinded to the utilized matrix system passed a waxed dental floss (Colgate Total) interproximally to assess PCP tightness. [16]

The optimum contact point is characterized by the ability of dental floss to pass through with minimal resistance, comparable to that of the opposing side’s natural teeth.Open PCPs allow dental floss to pass unobstructed, whereas PCPs are deemed tight if dental floss cannot be passed at all or shredded. .

### Radiographic assessment of proximal overhangs

**Fig. 3:**
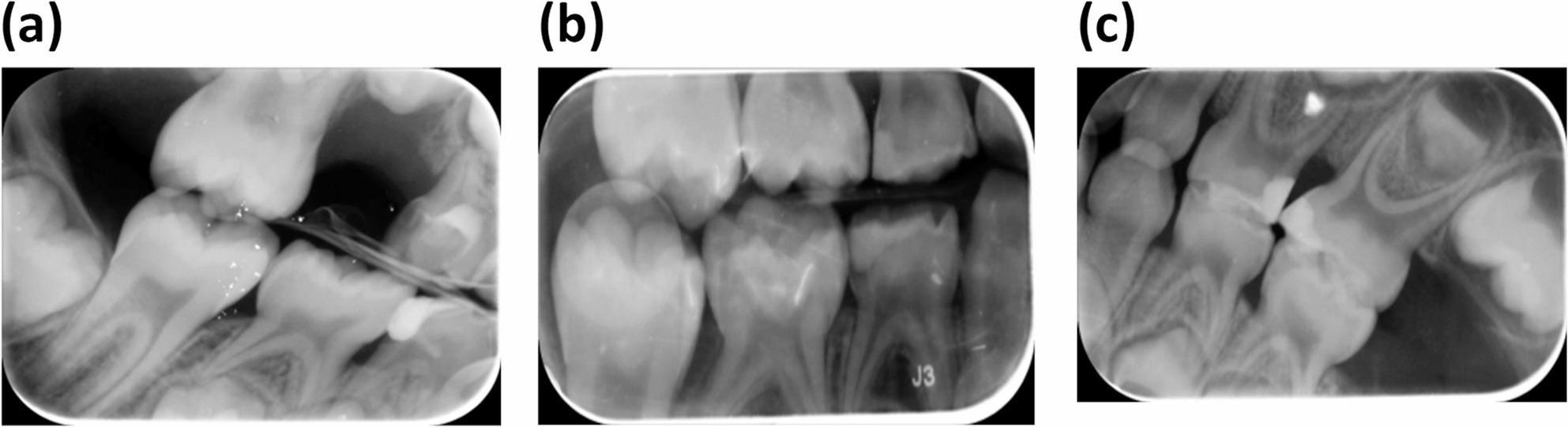
Bitewing radiographs showing postoperative assessment of the proximal overhangs of restoration (**a**) Positive Overhang (**b**) Negative Overhang (**c**) Absent Overhang

Two calibrated assessors, with 15 years of clinical experience, who were blinded about the type of matrix system, independently evaluated the.

digital bitewing radiographs to determine the proximal overhangs. [16, 28] The proximal overhangs were classified as positive when the excess of filling material extended beyond the cavity margin or healthy tooth structure at the proximal step of the restoration. If the filling material was found to be insufficient at the cavity boundary or typical tooth structure, it was categorized as a negative overhang. Conversely, the overhang was deemed absent when the filling material and tooth surface transitioned smoothly at the restoration’s proximal step (Figure:3). The assessment of intera-examiner and inter-examiner agreement was done utilizing the linear-weighted and unweighted kappa index. Two examiners, blinded to the experimental group, assessed 20 randomly selected restorations separately. One week later, they underwent a subsequent evaluation by an examiner. [29, 30]

respectively. These values demonstrated nearly perfect agreement [20, 21]. 

### Assessment operator ease and satisfaction

The questionnaire (Table 2) was given to the operator following the restoration of each proximal cavity in both groups. The assessment focused on the following aspects: ease of matrix band application and removal, trauma to gingival tissue during band application and removal, and dislodgment/displacement of restoration during the matrix band removal following cavity restorations [18]. The duration required for matrix system installation was measured utilizing a stopwatch, starting from the completion of pre-wedging to the full verification of the matrix system’s placement. [15]

**Table 2:** Questionnaire of operator ease and satisfaction

Q1	Ease of application of matrix band for the prepared cavity	(A) Easy	(B) Manageable	(C) Difficult
Q2	Ease of removal of matrix band after restoration	(A) Easy	(B) Manageable	(C) Difficult
Q3	Trauma to gingival tissue while applying and removing matrix band for restoration	(A) Present	(B) Absent	
Q4	Dislodgment/displacement of restoration while removing matrix band	(A) Yes	(B) No	
Q5	The amount of time needed to install the matrix system			

the validity of the questionnaire was determined by, conducting apilot study on 5 individuals for each group before the start of the study and The Cronbach alpha test was conducted. and it was found that the validity of the questionnaire was 0.86 which is higher than 0.70. Therefore, the design of questionnaires with high reliability was achieved.

### Assessment of patient comfort

**Fig. 4:**
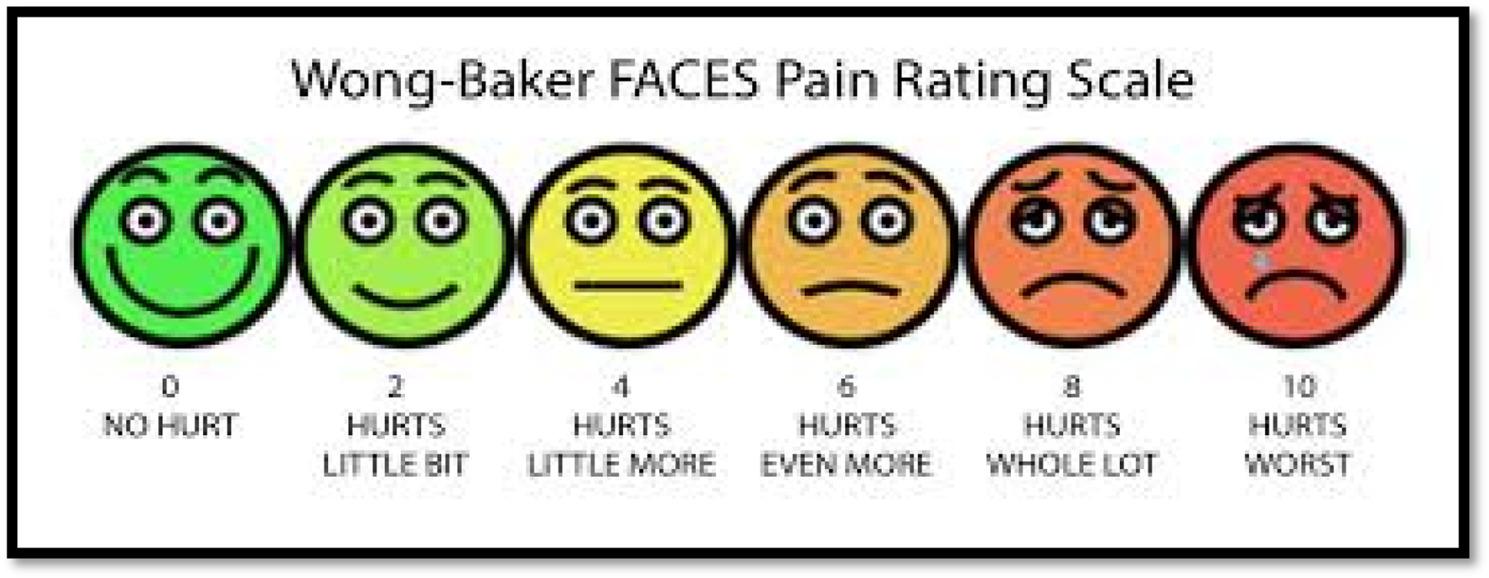
Wong Baker pain rating scale

The Wong-Baker pain rating scale was utilized to assess patient comfort [16]. (Fig. 4). The Wong-Baker Faces Pain Rating Scale is a pain assessment tool designed primarily for children but usable for anyone aged 3 and older. It consists of six cartoon faces ranging from a smiling face at 0, meaning “no hurt,” to a crying face at 10, which represents “hurts worst”. Each face is associated with a numeric value increasing in increments of 2 (0, 2, 4, 6, 8, 10) and a short phrase to help patients identify and communicate their level of pain by pointing to the face that best matches how they feel.

All collected data was calculated, tabulated, and statistically analysed using suitable statistical tests as follows.

1. A normality test was done to check normal distribution for data by Shapiro-Wilk test and all variables data showed Non-normal distribution.
2. Descriptive statistics was calculated in the form of Mean ± Standard deviation (SD), range (Max- Min). Qualitative data was presented as frequencies (n) and percentages (%).
3. The Chi-square test and Fisher exact was used to test significance of association between categorical variables.
4. *P* < 0.05 is considered significant.
5. Statistical analysis will be performed using the computer program SPSS software for windows version 26.0 (Statistical Package for Social Science, Armonk, NY: IBM Corp).

## Results

This study included 20 participants, consisting of 12 boys (60%) and 8 girls (40%). The comparison of gender distribution yielded a P-value of 0.3711, indicating no statistically significant difference (*P* > 0.05) between the proportions of boys and girls. The mean age of the boys was 6.8 ± 1.3 years, while the mean age of the girls was 7.1 ± 1.7 years. The overall age range for the sample was 5 to 9 years. The P-value for the age comparison was 0.749, which is also not statistically significant (*P *> 0.05). (Table 3) This study two distinct matrix systems used to restore class II cavities in primary posterior teeth. The data presented in Table (4) indicate a significant difference in PCPs between the FenderMate group and the T-band group (*p* = 0.006). The t-band matrix produced the highest number of optimal PCPs at 80%, subsequently followed by open PCPs at 15%, and tight PCPs at 5%. The FenderMate matrix system achieved a maximum of 55% open PCPs, followed by 30% optimum PCPs and 15% tight PCPs.

**Table 3:** Demographic data in this study

Boys					Girls						*P* value	
N		%			N			%				
12		60			8			40			0.3711	
		Age										
(mean ± SD)		6.8 ± 1.3			7.1 ± 1.7						0.749	
Min-max		5–9 years										
Tooth Location	55	54	64	65		75	74		84	85		Total
Tooth number	7	4	3	5		6	3		4	8		40

*Pvalue < 0.05: Significant

**Table 4:** Proximal contact points (PCPs) of both matrix systems used:

Proximal Contact Points (PCPs)	study groups				χ 2	p	Effect size	95% Confidence Interval
	FenderMate group n.20		T-band group (*n* = 20					
	n	%	n	%				
Optimum	6	30	16	80	10.1	0.006*	0.47 (large)	0.002–0.005
Open	11	55	3	15				
Tight	3	15	1	5				

*P value < 0.05: Significant

χ2=chi square test of significant

The kappa values indicating inter-examiner agreement were 0.87 for positive overhang, 0.89 for negative overhang, 0.94 for absent overhang, and for the intera-examiner -reliability, 0.98, 0.96, and 0.93,respectively. These values demonstrated nearly perfect agreement.

The proximal radiographic overhang assessment revealed no marked differences between the FenderMate and T-band groups (*p* = 0.26). Proximal overhangs were absent in 50% of restorations, negative in 30%, and positive in 20% when utilizing the FenderMate matrix. Proximal overhangs were absent in 75% of restorations, negative in 15%, and positive in 10% when utilizing the T-band matrix, as illustrated in Table (5).

**Table 5:** Radiographic assessment of proximal overhangs of both procedure modalities

Radiographical Assessment of Proximal Overhangs	study groups				χ 2	*p*	Effect size	95% Confidence Interval
	FenderMate group *n*.20		T-band group *n*.20					
	*n*.	%	*n*.	%				
Positive	4	20.0	2	10.0	2.67		0.33 (medium)	0.154-0.164
Negative	6	30.0	3	15.0		0.26		
Absent	10	50.0	15	75.0				

*P value *≥ 0.05: no significant

χ2 = chi square test of significant

**Fig. 5:**
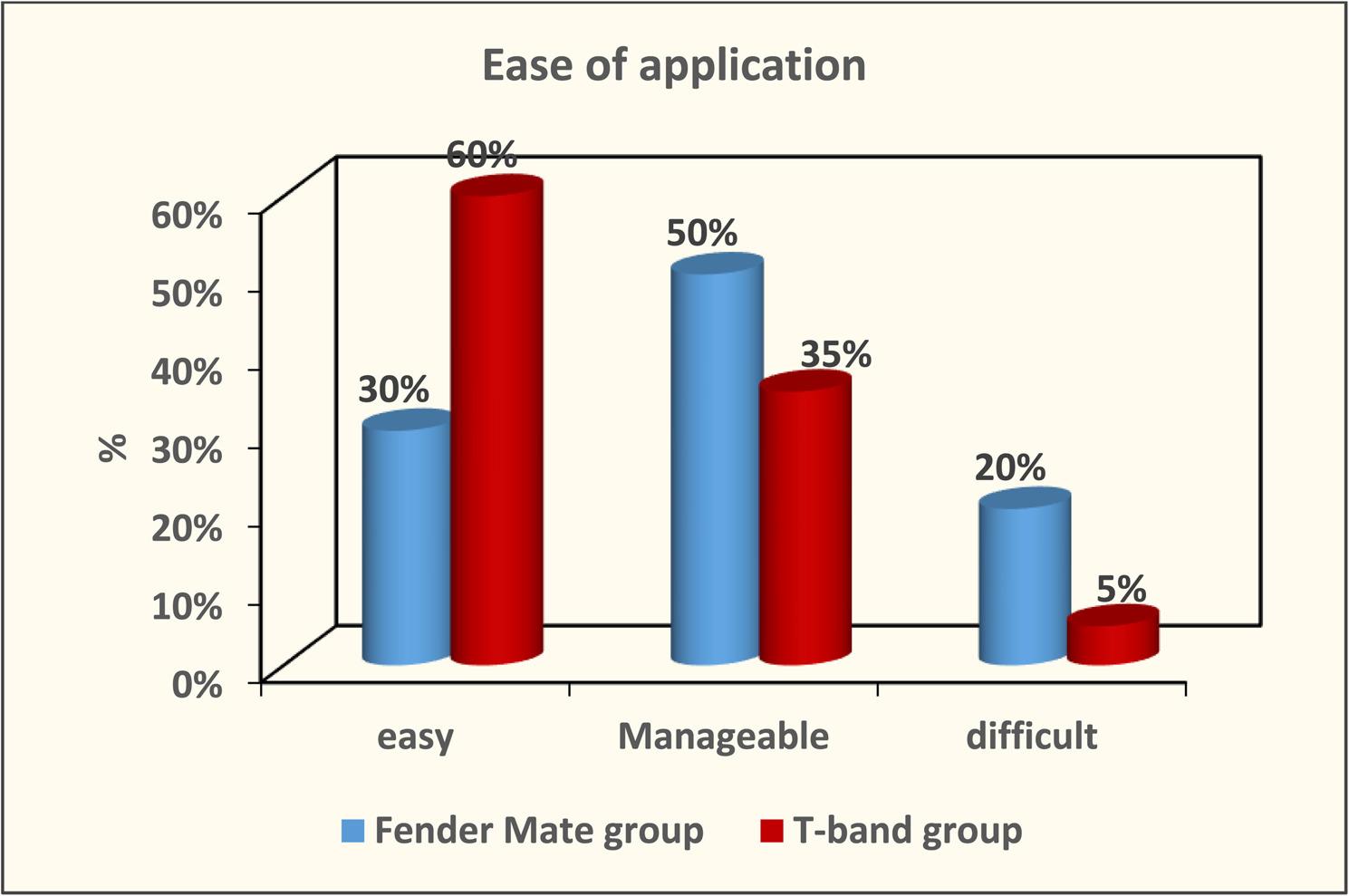
The percentage of ease of application of both matrix systems used (FenderMate matrix system and T-band matrix)

**Fig. 6:**
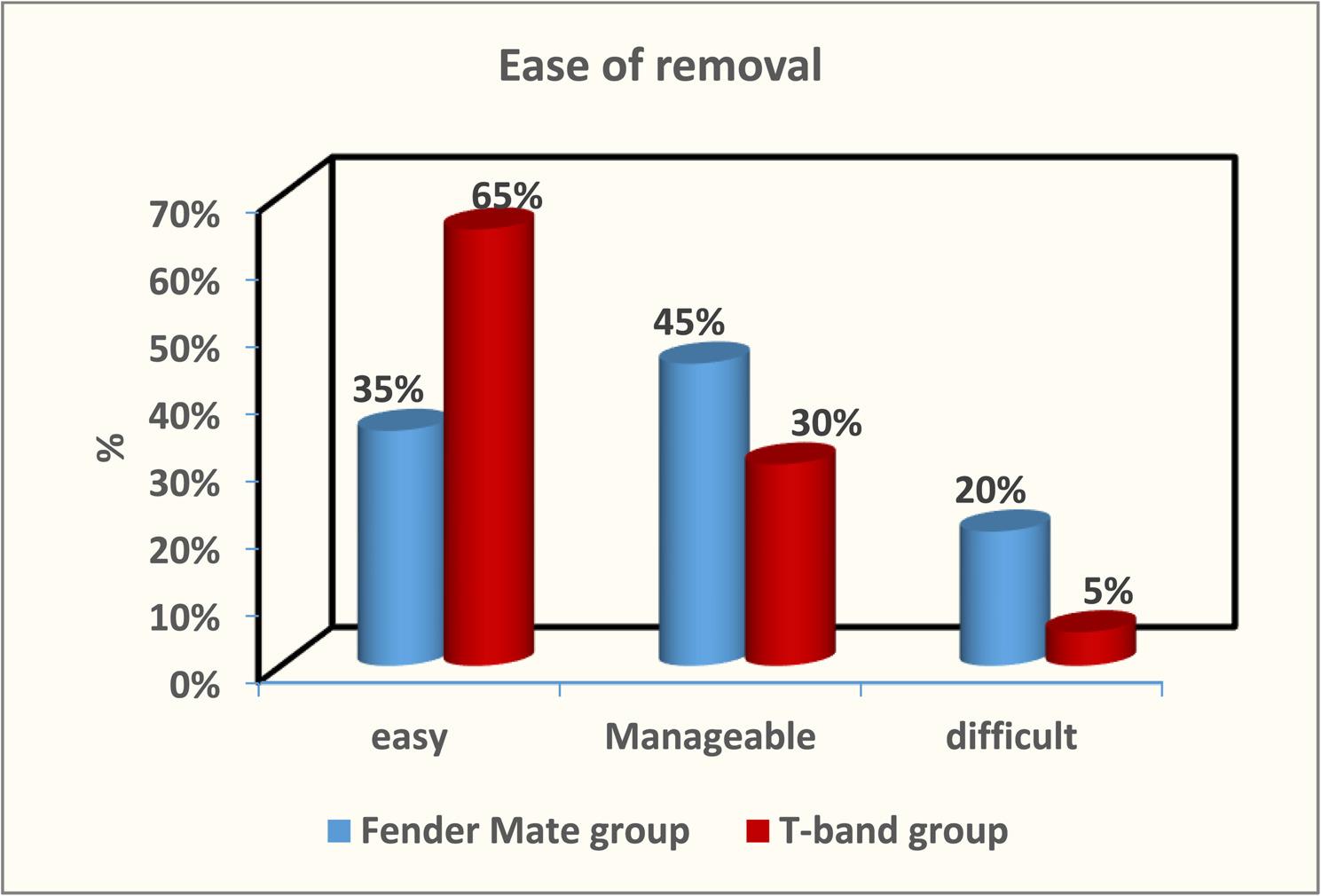
The percentage of ease removal of both matrix systems used (FenderMate matrix system and T-band matrix)

**Fig. 7:**
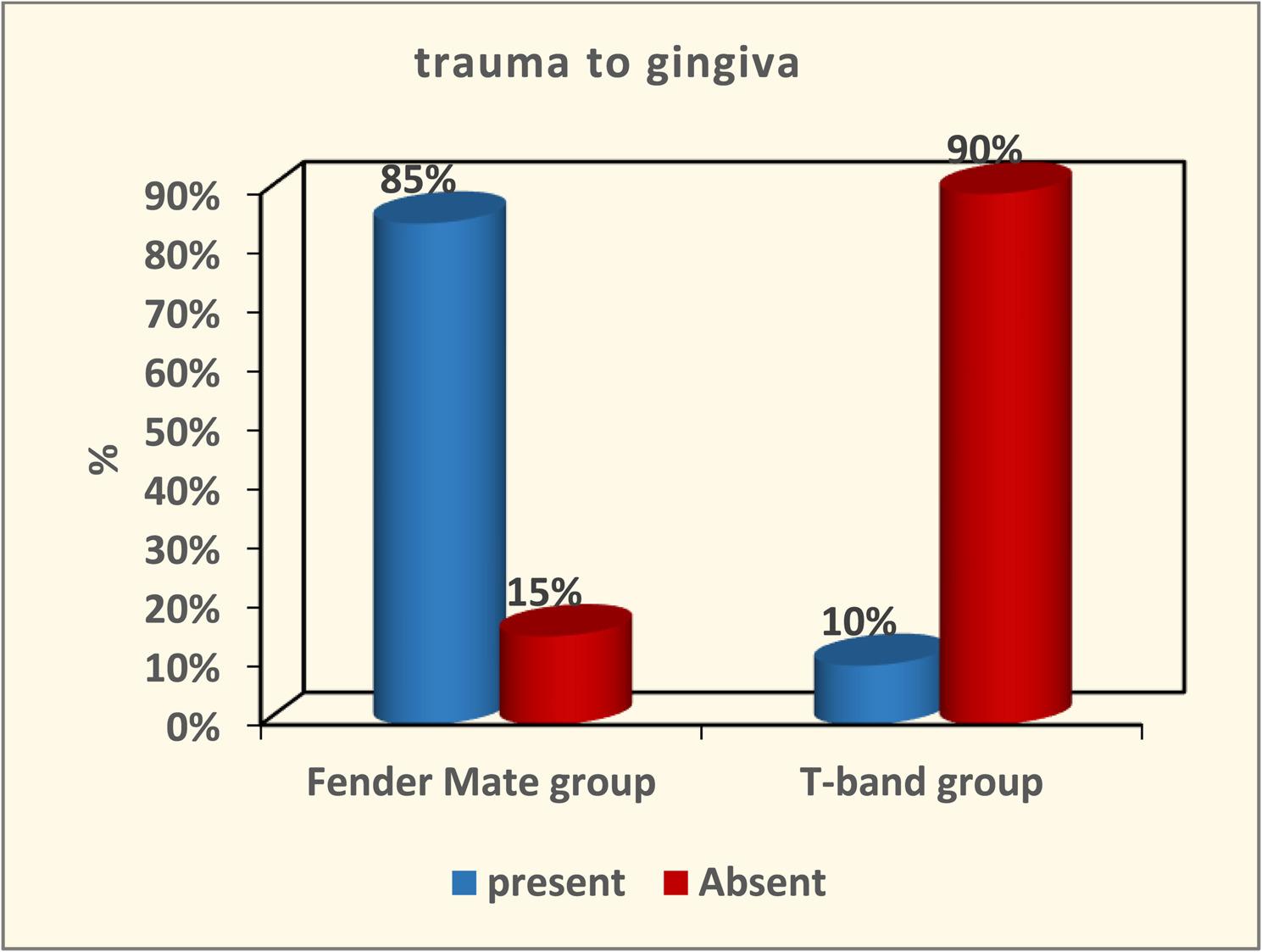
The percentage of gingival trauma of both matrix systems used (FenderMate matrix system and T-band matrix

**Fig. 8:**
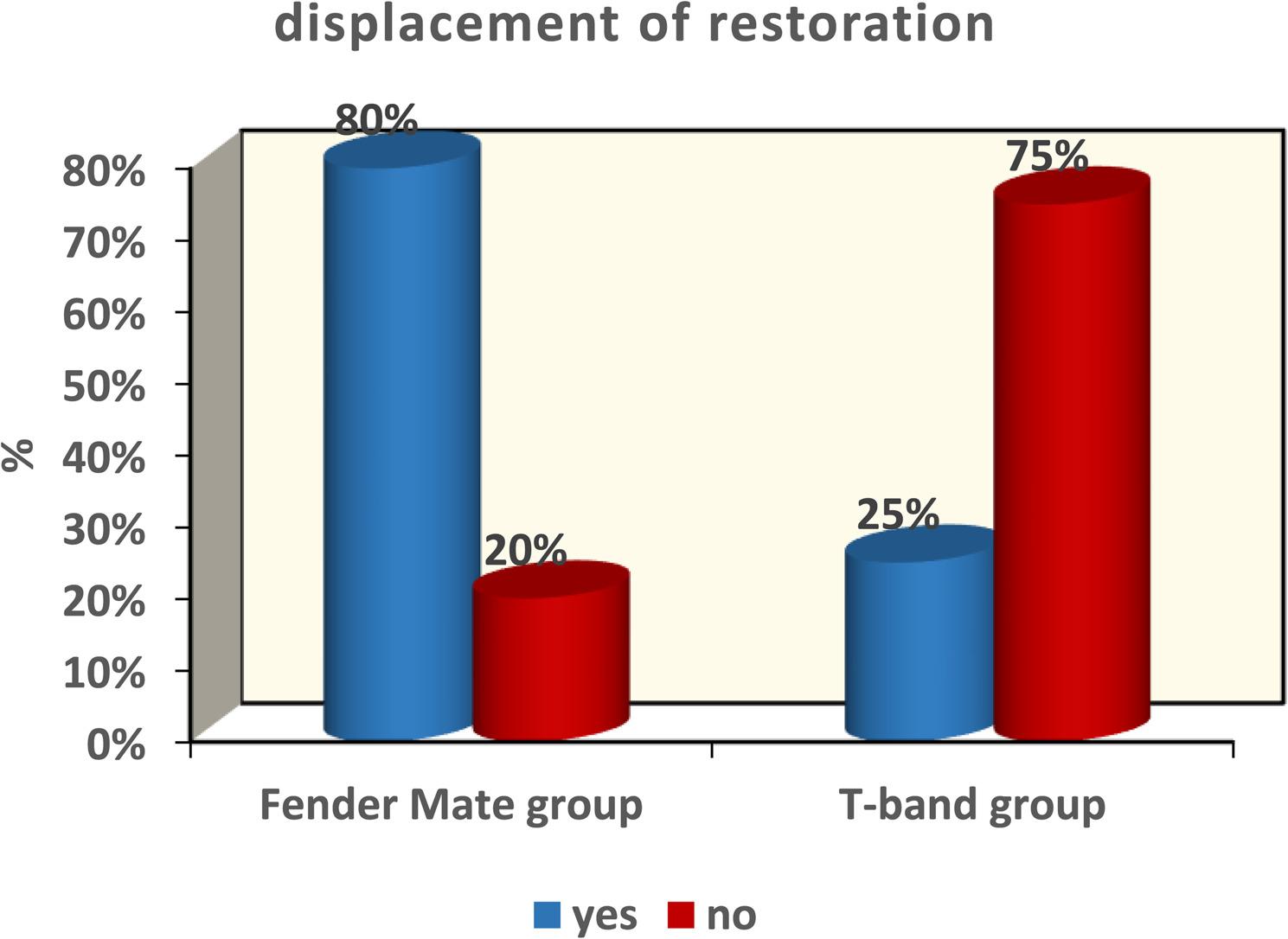
The percentage of dislodgment/displacement of restoration of both matrix systems used (FenderMate matrix system and T-band matrix)

**Fig. 9:**
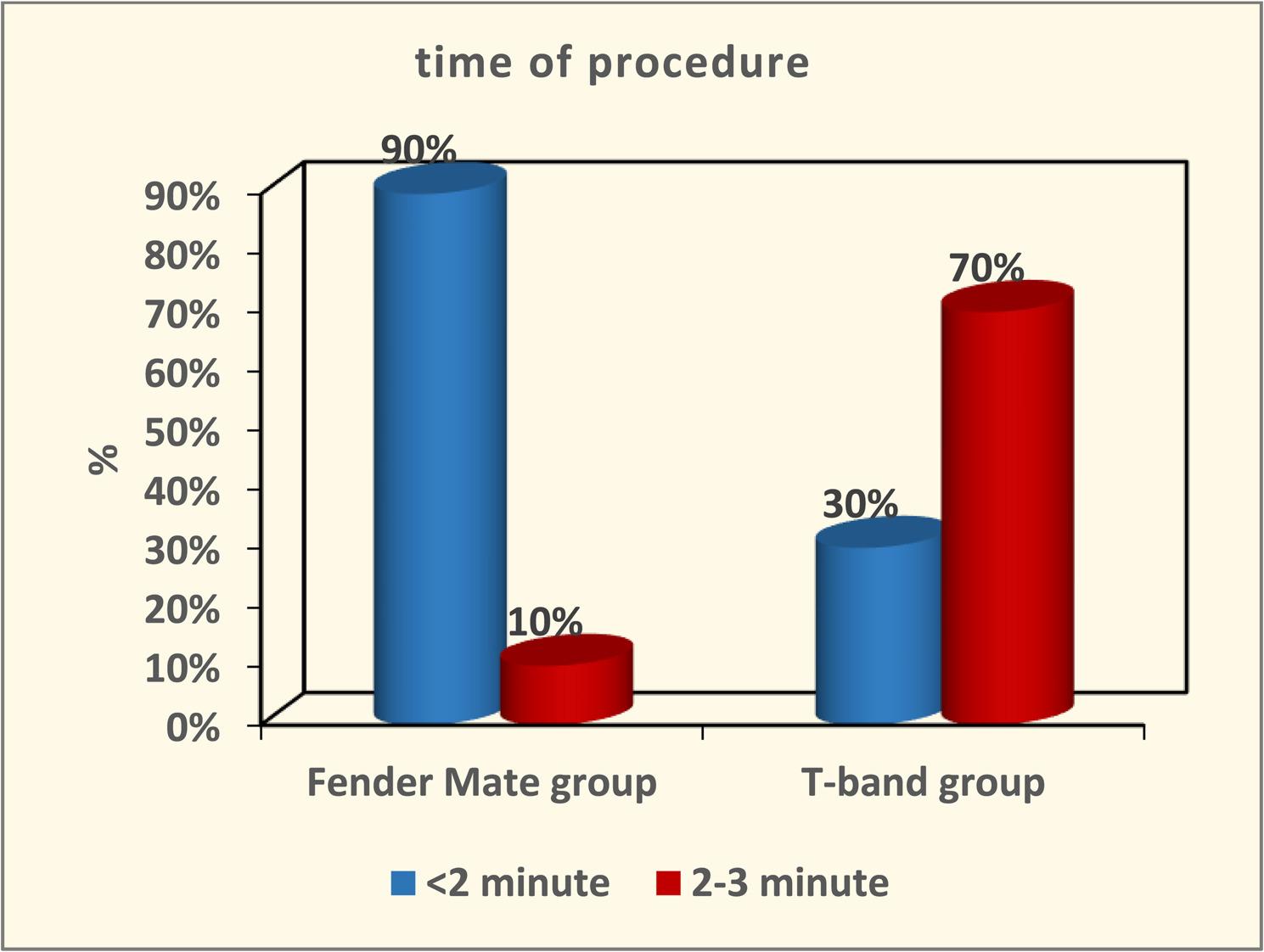
The percentage of consumed time of both matrix systems used (FenderMate matrix system and T-band marix)

The result of Cronbach alpha test conducted for the validity of the questionnaire was 0.86 which is higher than 0.70. Therefore, the design of questionnaires with high reliability was achieved. The result of this validated questionnaire showed no marked differences between the FenderMate and T-band groups concerning application ease or removal during class II cavity restoration in primary molars (*p* = 0.11 and *p* = 0.12, respectively) as depicted in Figure (5, 6). The FenderMate group exhibited a markedly greater frequency of gingival trauma and dislodgment/displacement of restoration compared to the T-band group (*p* = 0.0001) Figure (7, 8). In contrast, the time taken of the FenderMate procedure was considerably shorter than that of the T-band procedure (*p* = 0.0001), as depicted in Figure (9).

In relation to the Patient Comfort Score, the T-band group exhibited substantially greater percentages for scores of 0(no hurt) and 2(hurts little bit), at 30% and 55%, respectively, whereas the FenderMate group reported no patients with these scores. In contrast, a score of 6 (hurts even more) was considerably observed in the FenderMate group (60%), while the T-band group reported only 5%. Scores of 4(hurts little) more and 8 (hurts whole lot) were relatively diminished in both groups, with FenderMate recording 15% and 20%, respectively, while the T-band group achieved 10% for both scores. A score of 10(hurts worst) was minimally reported, with 5% in the FenderMate group and 0% in the T-band group. (Table (6) and Figure :10 )

**Table 6:** Patient comfort score of both matrix systems used:

Patient comfort score	study groups		χ 2	*p*	Effect size
	FenderMate group *n*.20	T-band group *n*.20			
	*N*(%)	*N*(%)			
0 (no hurt)	0(0.0)	6(30.0)	f	0.02*	1.0 (large)
2 (hurts little bit)	0(0.0)	11(55.0)	f	0.0001*	1.0 (large)
4 (hurts little)	3(15.0)	2(10.0)	f	0.99	0.2(Small)
6 (hurts even more)	12(60.0)	1(5.0)	13.8	0.0002*	0.85 (large)
8 (hurts whole lot)	4(20.0)	0(0.0)	f	0.106	1.0 (large)
10 (hurts worst)	1(5.0)	0(0.0)	f	0.99	1.0 (large)

**Fig. 10:**
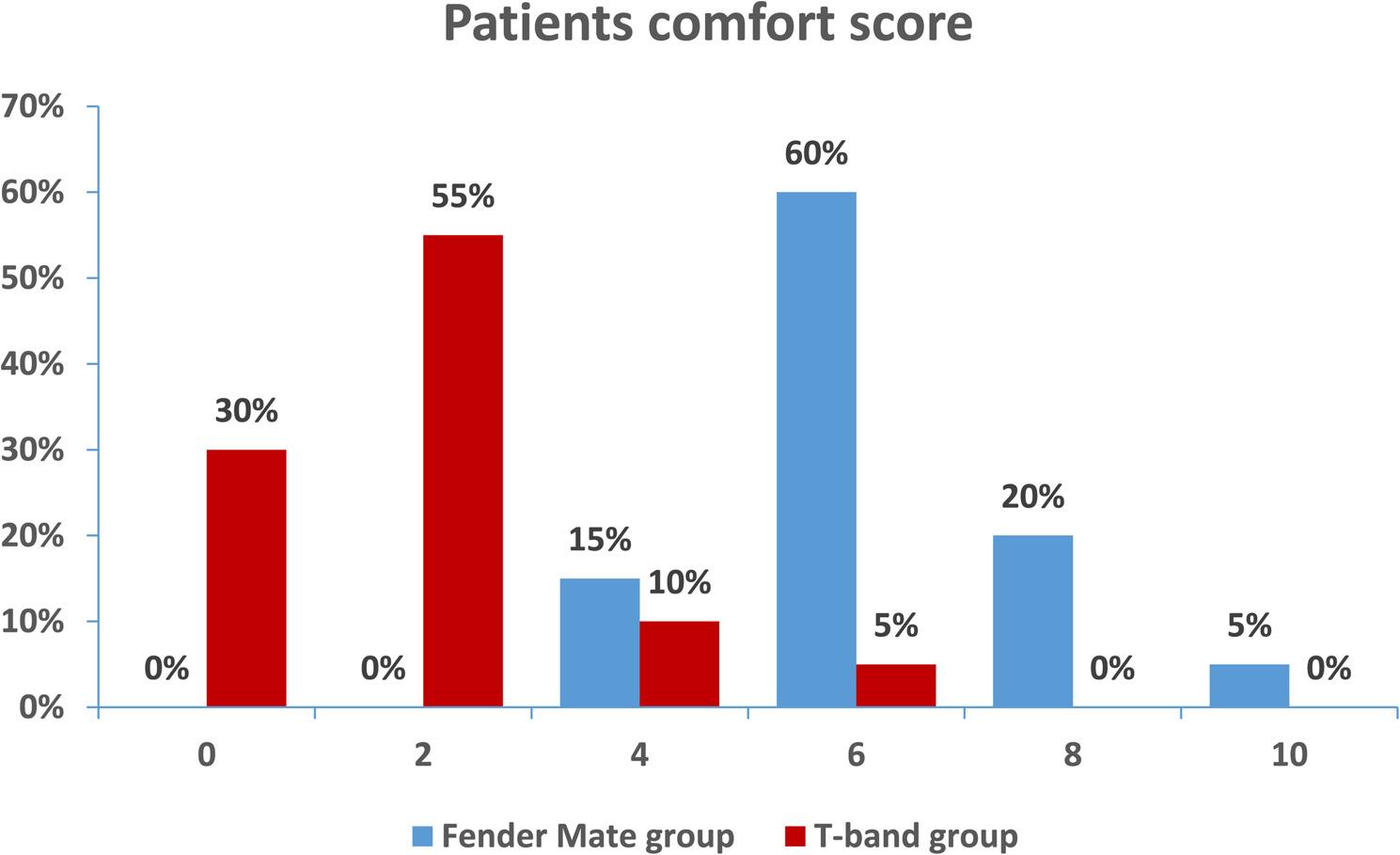
Patient's comfort score of both matrix systems used (FenderMate matrix system and T-band matrix)

## Discussion

Interproximal lesions in primary molars are more prevalent than occlusal lesions. proximal caries, in the mesial surfaces of the second primary molars and the distal surfaces of the first primary molars were observed with the highest frequency. [16, 30] The dentist’s primary objective is to restore aesthetics, function, and form of the tooth affected by proximal surface cavities. Well-formed, stable proximal contact can preserve gingival tissues while facilitating interdental cleaning, thus averting caries and contributing significantly to the preservation of dental arch integrity. [15, 18, 28].

A matrix band’s primary function is to compensate for absent walls and assist in containing the filler material. Numerous matrix band systems have been developed to restore the missing tooth structure [31]. This study utilized two different matrix methods to restore class II cavities: the FenderMate Prime system, which integrates a pre-curved sectional matrix band and wedge into a single device. [32] The T-band matrix is the most widely utilized matrix system in pediatric dentistry for class II restorations. [16]

This study selected the age group of 5–9 years, as the first permanent molar generally erupts at approximately 6 years of age. Upon eruption of this molar, physiological spaces close, inter-tooth contacts tighten, and the risk of dental caries escalates. [15]

This study employed a split-mouth technique conducted within the same arch to eliminate discrepancies in mean operating time between the two quadrants. A split-mouth study is advantageous as it minimizes intra-individual variability in the impact of treatment estimations [33]. 

The current investigation utilized waxed dental floss to assess proximal contact tightness, as it is the most widely employed method for this evaluation [34]. The findings of this study indicate that the FenderMate matrix exhibited a higher percentage of open contact points (55%) than the T-band matrix (15%). This can be due to the challenges associated with the FenderMate Prime matrix in bending and conforming to the tooth. Additionally, this system’s wedging effect may have been responsible for the increased number of open contacts. This finding aligns with Patel et al. [16] , who determined that the FenderMate system exhibited the most open contact points relative to the Tofflemire matrix and Unimatrix R.

In contrast, the study by Dindukurthi et al. [18] reported that the FenderMate system did not exhibit any open or defective contacts. The finding of this study differs from the systematic review by Kamble et al., [20] which concluded that the sectional matrix band system was more effective than the circumferential system in achieving optimal proximal contacts in class II posterior composite restorations. These discrepancies may be attributed to the different matrix systems evaluated, sectional matrices with separation rings produced significantly tighter contacts, and the focus on permanent teeth in the studies. Moreover, an in-vtro study by Gigova et al. (2025) found that using sectional matrix systems specifically designed for primary teeth (MuJuniorKit) significantly improves the proximal contacts of Class II composite restorations in primary molars. Additionally, the study reported that circumferential matrices are associated with a higher risk of edge formation compared to sectional matrices. This difference in findings may be attributed to the larger sample size used by Gigova et al., [35] which involved 240 s primary molars.

This study evaluated proximal overhangs utilizing digital bitewing radiographs, as the visual examination of the cervical areas for marginal defects such as overhangs, ditches, and gaps is hindered by the presence of gingival tissue and adjacent teeth [36]. This study revealed insignificant differences in overhang formation between the two matrix modalities. The placement of matrix bands and wedges may influence the formation of overhangs more significantly than other factors [37]. None of the bands utilized in this study successfully prevented the occurance of incorrect proximal contours, as documented in the existing literature [37, 38]. This finding aligns with Dindukurthi et al. [18], who detected insignificant differences in overhang formation among FenderMate, ProMatrix, and T-band matrix. On the other hand, a study by Abdelaziz et al. [39] reported significantly higher percentages of overhang-free restorations using sectional matrix systems (TOR VM and Composi-Tight 3D Fusion™) compared to the circumferential matrix system (Tofflemire Matrix Retainer group). This discrepancy may be attributed to differences in the matrix systems used and the fact that their study was conducted on permanent teeth, whereas ours focused on deciduous teeth.

The examination showed that the T-band matrix system non-significantly surpassed the FenderMate system in ease of application and removal for cavity restorations. This difference is attributed mainly to the thickness of the band material and the additional application of a wedge, which are applied separately, for band stabilization in the T-band system. FenderMate, which integrates the band and wedge in one unit, was more difficult to apply and remove, likely causing more trauma to gingival tissue and resulting in more displacement or dislodgment of restorations during removal. The design of FenderMate (a notch for contouring) also contributed to this difference [18]. This finding is not aligned with the systematic review by Kambel et al.[20]. which reported that sectional matrix was considered easier to use.

The FenderMate matrix system caused more trauma to gingival tissue during both the matrix application and removal than the T-band system. The short cervical-occlusal crown length in primary teeth may contribute to challenges in placing matrix bands. [40] The T-band allows for adjustable placement depth of the matrix band and wedge based on crown height, a feature not available in the FenderMate matrix system, where the matrix band and wedge are fixed together. In this scenario, the band and wedge may exert pressure on the gingival tissue, resulting in increased trauma. In addition, the degree of keratinization, position, and shape of interdental gingiva may contribute to trauma during matrix band placement, in contrast to adult gingiva, which exhibits greater keratinization. This finding agrees with Dindukurthi et al. [18], who determined that the FenderMate matrix caused greater trauma to gingival tissue during the matrix band application and removal compared to Pro-Matrix and T-band.

The fenderMate matrix system exhibited greater dislodgment and displacement of restorations during band removal than the T-band. This may result from a notch in the FenderMate matrix system, which facilitates contour creation during proximal cavity restoration. The T-band was plain and lacked a notch, which may account for the reduced dislodgment/displacement of restorations during application. This finding aligns with the conclusions of Dindukurthi et al. [18], who determined that FenderMate resulted in greater displacement or dislodgment of restorations during band removal compared to Pro-Matrix and T-band.

The FenderMate system required less time for placement compared to the T-band. This can be attributed to the distinctive characteristic of the FenderMate matrix system, in which the matrix and wedge coexist as a single unit. This finding aligns with Patel et al. [16], who determined that the FenderMate system required the least time for placement compared to Unimatrix R sectional and Tofflemire systems.

The Wong-Baker Faces Pain Rating Scale was utilized to assess patient comfort, as it serves as a subjective measure reliant on the child’s contextual, emotional, and sensory conditions. It demonstrated strong construct validity and is regarded as one of the most dependable self-report measures of pain, integrating visual and numerical elements. This scale is straightforward and user-friendly, contributing to its widespread application in assessing pain in children [41–43]. The T-band group demonstrated superior patient comfort compared to the FenderMate system. Patients in the FenderMate group reported increased discomfort attributed to the wedge-shaped design of the FenderMate Prime matrix, which induced pain upon insertion into the interdental space. This phenomenon can be attributed to the short cervical-occlusal crown length of primary teeth and the ability to adjust the matrix band and wedge placement depth according to crown height using T-band systems, unlike the FenderMate system, which features an inseparable assembly of wedge and matrix. This may result in the gingival tissue impingement, leading to gingival trauma and subsequent discomfort for the child. [18] This finding aligns with the conclusions of Patel et al. [16], who determined that the FenderMate system resulted in greater patient discomfort than the Unimatrix R sectional and Tofflemire systems.

The study results are limited by: the lack of extensive literature regarding matrix systems for Class II cavity restoration in primary molars, the small sample size and the lack of follow-up. None of the matrix systems assessed accomplished an optimal proximal contour for primary molars. This underscores the necessity for additional in vitro and in vivo research with larger sample size. The study’s exclusion and inclusion criteria could have been more comprehensive by incorporating additional relevant variables, including cavity depth, location, and size, as these factors may impact the results. Radiographic assessment was not digitized using the latest software.Longitudinal follow up will be highly recommended to highlight changes over time.

These findings might assist clinicians in the futur selection of matrix system in pediatric Class II restorations.

## Conclusions

the results of this study, within the sample size and age range mentioned earlier, concluded that:

- The T-band demonstrated signicantly enhanced efficacy in establishing appropriate proximal contacts (*p* = 0.006).
- The T-band produced unsignicantly fewer overhangs than the FenderMate system (*p* = 0.26).
- In contrast, the time taken of the FenderMate procedure was considerably shorter than that of the T-band procedure (*p* = 0.0001).
- The T-band demonstrated nonsignificant ease of application and removal, (*p* = 0.11 and *p* = 0.12, respectively) resulting in minimal trauma to the gingival tissue (*p* = 0.0001).
- Significant increased dislodgment and displacement of restorations were observed with the FenderMate matrix system during the removal of bands(*p* = 0.0001) .
- The FenderMate matrix system demonstrated greater time efficiency than the T-band system(*p* = 0.0001) .
- The T-band group demonstrated superior patient comfort relative to the FenderMate system.

## Data Availability

Datasets of the current study are available from the first author (Abdou N) upon request.
